# Microsatellite marker development by partial sequencing of the sour passion fruit genome (*Passiflora edulis* Sims*)*

**DOI:** 10.1186/s12864-017-3881-5

**Published:** 2017-07-21

**Authors:** Susan Araya, Alexandre M Martins, Nilton T V Junqueira, Ana Maria Costa, Fábio G Faleiro, Márcio E Ferreira

**Affiliations:** 1 Department of Agronomy, Campus Universitário Darcy Ribeiro, University of Brasilia (UnB), Brasília, 70910-900 Brazil; 2 Embrapa Genetic Resources and Biotechnology, Genetics Laboratory, CEP 70770-917, Brasilia, DF Brazil; 3 Embrapa Cerrados, Caixa Postal 08233, CEP, Planaltina, DF 73310-970 Brazil; 4 Embrapa Labex USA, Agricultural Research Service, USDA, Bldg. 006 Rm. 200 10300 Baltimore Ave, Beltsville, MD 20705 USA

**Keywords:** De novo genome assembly, Microsatellite, Cross-species transferability

## Abstract

**Background:**

The *Passiflora* genus comprises hundreds of wild and cultivated species of passion fruit used for food, industrial, ornamental and medicinal purposes. Efforts to develop genomic tools for genetic analysis of *P. edulis*, the most important commercial *Passiflora* species, are still incipient. In spite of many recognized applications of microsatellite markers in genetics and breeding, their availability for passion fruit research remains restricted. Microsatellite markers in *P. edulis* are usually limited in number, show reduced polymorphism, and are mostly based on compound or imperfect repeats. Furthermore, they are confined to only a few *Passiflora* species. We describe the use of NGS technology to partially assemble the *P. edulis* genome in order to develop hundreds of new microsatellite markers.

**Results:**

A total of 14.11 Gbp of Illumina paired-end sequence reads were analyzed to detect simple sequence repeat sites in the sour passion fruit genome. A sample of 1300 contigs containing perfect repeat microsatellite sequences was selected for PCR primer development. Panels of di- and tri-nucleotide repeat markers were then tested in *P. edulis* germplasm accessions for validation. DNA polymorphism was detected in 74% of the markers (PIC = 0.16 to 0.77; number of alleles/locus = 2 to 7). A core panel of highly polymorphic markers (PIC = 0.46 to 0.77) was used to cross-amplify PCR products in 79 species of *Passiflora* (including *P. edulis*), belonging to four subgenera (*Astrophea, Decaloba, Distephana* and *Passiflora*). Approximately 71% of the marker/species combinations resulted in positive amplicons in all species tested. DNA polymorphism was detected in germplasm accessions of six closely related *Passiflora* species (*P. edulis*, *P. alata, P. maliformis, P. nitida, P. quadrangularis* and *P. setacea*) and the data used for accession discrimination and species assignment.

**Conclusion:**

A database of *P. edulis* DNA sequences obtained by NGS technology was examined to identify microsatellite repeats in the sour passion fruit genome. Markers were submitted to evaluation using accessions of cultivated and wild *Passiflora* species. The new microsatellite markers detected high levels of DNA polymorphism in sour passion fruit and can potentially be used in genetic analysis of *P. edulis* and other *Passiflora* species.

**Electronic supplementary material:**

The online version of this article (doi:10.1186/s12864-017-3881-5) contains supplementary material, which is available to authorized users.

## Background

*Passiflora* is a highly diverse genus with approximately 520 species distributed in tropical regions of America, Asia and Africa [1]. Despite taxonomical uncertainties, approximately 96% of the *Passiflora* species are found in South and Central America [2]. Major centers of diversity include regions of Brazil and Colombia [3, 4], both countries with hundreds of species catalogued. However, just a few *Passiflora* species are used in agriculture, mostly for production of fruits, which are consumed *in natura* or as juice. Passion fruit species are also used as ornamentals, in the food industry and for medicinal purposes.

Sour passion fruit (*P. edulis*) is by far the most important commercial *Passiflora* species worldwide. It is an allogamous species, displaying a well documented variability of shapes and colors of fruits, flowers and plants. Genetic diversity in *P. edulis* has been assessed by morphological descriptors [5–7] and agronomic traits [8–10]. Detection of DNA polymorphism in *P. edulis* has been pursued with different types of molecular markers, such as Inter Simple Sequence Repeat (ISRR) [11], Random Amplified Polymorphic DNA (RAPD) [12–14], Amplified Fragment Length Polymorphism (AFLP) [15, 16] and microsatellites [17–19]. High levels of genetic variability have been recorded in morphological and agronomic evaluations of sour passion fruit, as well as in most marker systems. However, the use of microsatellite markers in genetic analysis of *P. edulis* underscores low DNA polymorphism [16–18] in an otherwise highly diverse species.

Advantages of microsatellite markers over other technologies include high reproducibility, co-dominance, high polymorphic information content (PIC) and multi-allelism [20–22]. Less than 200 microsatellite markers have been developed for *P. edulis* [17, 19, 23] and only a small fraction of these markers have been validated and used in genetic studies [16, 23–25]. The few polymorphic *P. edulis* microsatellite markers are based on compound or imperfect motifs, which are hard to interpret on routine genotyping assays due to allele binning difficulties [26, 27]. This could be a constraint to some applications, especially for population genetic studies [28, 29]. Perfect microsatellite markers (i.e. repeat of the same nucleotide motif without interruption or variation) would be more suitable, but they are only a small fraction (~10%) of the total number of *P. edulis* markers [17, 19, 23]. Also, the use of microsatellite markers in *Passiflora* has been limited to a few species, such as *P. edulis* [17, 19, 23], *P. alata* [30, 31], *P. cincinnata* [18, 19], *P. setacea* [19], and *P. contracta* [32]. This is only a tiny fraction (~1%) of the known *Passiflora* species. Similar constraints to microsatellite marker availability and use are also observed in other *Passiflora* species. Therefore, although there is a wide number of applications of microsatellite markers in genetics and breeding, their development and availability for passion fruit research is still restricted.

Microsatellite detection and isolation has been most often based on enrichment of genomic libraries by selective hybridization [33] or by primer extension [34]. Another approach is to identify microsatellite repeats in DNA databases such as EST sequences [35]. The development of microsatellite markers in *Passiflora* has been based on the construction of genomic libraries enriched for simple sequence repeats [17–19, 23, 30–32]. This is an effective but time and labor consuming technique that can lead to microsatellite discovery and marker development. However, new approaches such as next-generation sequencing (NGS) can provide a large number of high quality genome sequences that can be obtained faster and at reduced costs, facilitating the detection of thousands of microsatellite sites in the genome of a target species [36–39].

In the present study we used NGS to sequence the *P. edulis* genome. We then screened contig sequences obtained by partial de novo assembly to detect perfect microsatellite sites. This data was used to develop and validate microsatellite markers using *P. edulis* accessions of the germplasm bank. Markers were then evaluated for quality and polymorphism in *P. edulis* and five closely related *Passiflora* species and, also, for cross-species transferability to 78 *Passiflora* species belonging to four subgenera (*Astrophea, Decaloba, Distephana and Passiflora*), recently collected in Brazil.

## Methods

*DNA extraction and genome sequencing –* Fresh young leaves of the accession *Passiflora edulis* CPGA1, a sour yellow rind commercial cultivar of passion fruit, were used for DNA extraction with the standard CTAB protocol [40]. The construction of the genomic DNA fragment library and massive parallel paired-end sequencing by synthesis using an Illumina GAII sequencer followed the Illumina protocol.

De novo *genome assembly* – The presence of non-nuclear and/or exogenous DNA sequences on the passion fruit DNA database was verified by BLASTing it against a database of chloroplast, mitochondrial and potential contaminant DNA (fungi, bacteria and virus). Extraneous sequences were removed from the analysis. The short-read correction tool of SOAP*denovo* (Release 1.05), used to correct Illumina GA reads for large plant and animal genomes [41], was applied to FASTQ formatted files containing DNA sequencing reads. The CLC trimmer function (default limit = 0.05) (CLC Genomics Workbench 4.1 software, CLC Bio, Aarhus, Denmark) was then used to eliminate Illumina sequencing adapters and low quality reads. ErrorCorrection routines and KmerFreq were run with default parameters (seed length = 17, quality cutoff = 5). Final FASTQ files were submitted to de novo assembly routines using a bubble size of 50 bp on the CLC Genomics Workbench (Assembly Length Fraction = 0.5; Similarity = 0.8), followed by a scaffolding procedure by MipScaffolder [42]. Mismatch, deletion and insertion cost parameters were set to 2, 3 and 3, respectively. The k-mer size on CLC Bio assembler was set to 25 bp and the coverage cutoff to 10X. During assembly, the default word length parameter was adjusted to 25, using k-mer (de Bruijn graph k-mer) overlap information in order to assure unambiguous paths of resulting contigs. The fraction of short insert size contigs >160 bp was considered in the analysis. Overlaps between sequences were depicted by de Bruijn graph structures [43].

*Identification of microsatellite sites and marker development* – The partial de novo sequence assembly results were submitted to simple sequence repeat loci identification using PHOBOS [44]. The location and number of di-, tri-, and tetra-nucleotide SSRs in the draft de novo genome assembly were listed and quantified. Sequence repeats located in putative coding regions were identified with the gene model version TAIR 9 using *P. edulis* contigs blasted against *Arabidopsis thaliana* transcripts (AtGDB171). An ab initio prediction of coding regions was also performed using *geneid* [45] [http://genome.crg.es/software/geneid/]. Both analyses were considered for the selection of microsatellites located in structural and coding regions. Only microsatellite sites located in genomic regions with minimum 15X coverage were considered for marker development. A database of simple sequence repeats with four or more di-nucleotide repeats and three or more tri- and tetra-nucleotide repeats was created. Microsatellite loci showing a simple motif exactly repeated in tandem (“perfect microsatellite”) were listed and those with compound (more than one motif) or imperfect repeats were set aside. Perfect microsatellites with minimum 3× motif repeat and located on contigs with minimum 2.5 Kb length and 20X average coverage, as an attempt to maximize loci independence and marker quality, composed the group of selected markers. Finally, PCR primer pairs for 816 perfect microsatellite loci were developed with Primer3Plus [46].

*Plant materials and microsatellite marker descriptive statistics –* Ten accessions of sour passion fruit (*P. edulis* Sims), maintained by the Passion Fruit Germplasm Bank, Embrapa Cerrados, Planaltina, DF, were used to evaluate if the new set of markers is suitable for genetic analysis of passion fruit. Passport data of the passion fruit accessions used in the present study is described on Table 1 (rows 1 to 10). These accessions represent a diverse group of cultivars and local varieties collected in different regions of Brazil. The only exception is accession “Gulupa” from Colombia. This accession, however, is believed to have been originally collected in Brazil and later introduced in Colombia [47, 48] and was, therefore, also used in the analysis. These ten *P. edulis* accessions were genotyped with a random sample of 60 di- and tri-nucleotide microsatellites. Marker polymorphism, number of alleles, heterozigosity, PIC values and other statistics were estimated by CERVUS [49].

**Table 1:** Germplasm accessions of passion fruit (*Passiflora* spp.) collected in different regions of Brazil and genotyped with the new microsatellite markers

	Species	Subgenus	Origin
1	*Passiflora edulis* Sims	*Passiflora*	Selection Embrapa CPGA1, Distrito Federal
2	*Passiflora edulis* Sims	*Passiflora*	Selection Embrapa CPMSC1, Paraná
3	*Passiflora edulis* Sims	*Passiflora*	Selection Maguary, Minas Gerais
4	*Passiflora edulis* Sims	*Passiflora*	Cafuringa, Distrito Federal
5	*Passiflora edulis* Sims	*Passiflora*	Niquelândia, Goiás
6	*Passiflora edulis* Sims	*Passiflora*	Oliveira, Minas Gerais
7	*Passiflora edulis* Sims	*Passiflora*	Búzios, Rio de Janeiro
8	*Passiflora edulis* Sims	Passiflora	Criciúma, Santa Catarina
9	*Passiflora edulis* Sims	*Passiflora*	Jundiaí, São Paulo
10	*Passiflora edulis* Sims	*Passiflora*	Gulupa, Colombia (originally from Brazil)
11	*Passiflora edulis* Sims	*Passiflora*	BRS Maracujá Jaboticaba, Distrito Federal
12	*Passiflora actinia* Hook.	*Passiflora*	Curitiba, Paraná
13	*Passiflora acuminata* DC.	*Passiflora*	Manaus, Amazonas
14	*Passiflora alata* Curtis	*Passiflora*	Monte Verde, Minas Gerais
15	*Passiflora alata* Curtis	*Passiflora*	Selection Embrapa, Distrito Federal
16	*Passiflora alata* Curtis	*Passiflora*	Selection Embrapa, Distrito Federal
17	*Passiflora alata* Curtis	*Passiflora*	Selection Embrapa, Distrito Federal
18	*Passiflora alata* Curtis	*Passiflora*	Trancoso, Bahia
19	*Passiflora ambigua* Hemsl.	*Passiflora*	Confresa, Mato Grosso
20	*Passiflora amethystina* Mikan	*Passiflora*	Monte Verde, Minas Gerais
21	*Passiflora araujoi* Sacco	*Distephana*	Santarém, Pará
22	*Passiflora auriculata* Kunth	*Decaloba*	Manaus, Amazonas
23	*Passiflora bahiensis* Klotzsch	*Passiflora*	Lençóis, Bahia
24	*Passiflora biflora* Lam.	*Decaloba*	Novo Airão, Amazonas
25	*Passiflora boticarioana* Cervi	*Passiflora*	Conceição do Mato Dentro, Minas Gerais
26	*Passiflora caerulea* L.	*Passiflora*	Bento Gonçalves, Rio Grande do Sul
27	*Passiflora capsularis* L.	*Decaloba*	Planaltina, Distrito Federal
28	*Passiflora cerasina* Annonay & Feuillet	*Passiflora*	Presidente Figueiredo, Amazonas
29	*Passiflora cerradense* Sacco	*Astrophea*	Planaltina, Distrito Federal
30	*Passiflora cervii* Milward-de-Azevedo	*Decaloba*	Caeté, Minas Gerais
31	*Passiflora chlorina* L. K. Escobar	*Astrophea*	Caeté, Minas Gerais
32	*Passiflora cincinnata* Mast.	*Passiflora*	Rio Pardo de Minas, Minas Gerais
33	*Passiflora coccinea* Aubl.	*Passiflora*	Pontes e Lacerda, Mato Grosso
34	*Passiflora decaisneana* G. Nicholson	*Passiflora*	Planaltina, Distrito Federal
35	*Passiflora edmundoi* Sacco	*Passiflora*	Rio Pardo, Minas Gerais
36	*Passiflora eichleriana* Mast.	*Passiflora*	Criciúma, Santa Catarina
37	*Passiflora elegans* Mast.	*Passiflora*	Patos de Minas, Minas Gerais
38	*Passiflora ferruginea* Mast.	*Decaloba*	Rio Branco, Acre
39	*Passiflora foetida* L.	*Passiflora*	Belém, Pará
40	*Passiflora galbana* Mast.	*Passiflora*	Ponte Nova, Minas Gerais
41	*Passiflora gardneri* Mast.	*Passiflora*	Silvania, Goiás
42	*Passiflora gibertii* Brown	*Passiflora*	Poconé, Mato Grosso
43	*Passiflora glandulosa* Cav.	*Passiflora*	Igarapé-açú, Pará
44	*Passiflora haematostigma* Mart. ex Mast.	*Astrophea*	Natividade, Tocantins
45	*Passiflora hatschbachii* Cervi	*Passiflora*	Jaíba, Minas Gerais
46	*Passiflora hypoglauca* Harms	*Passiflora*	Ouro Preto, Minas Gerais
47	*Passiflora incarnata* L.	*Passiflora*	Centroflora, Botucatu, São Paulo
48	*Passiflora jilekii* Wawra	*Passiflora*	Manhuaçu, Minas Gerais
49	*Passiflora junqueirae* Imig & Cervi	*Passiflora*	Caparaó, Minas Gerais
50	*Passiflora kermesina* Link & Otto	*Passiflora*	São José do Laranjal, Minas Gerais
51	*Passiflora laurifolia* L.	*Passiflora*	Picos, Piauí
52	*Passiflora ligularis* Juss.	*Passiflora*	Commercial Orchard
53	*Passiflora loefgrenii* Vitta	*Passiflora*	Criciúma, Santa Catarina
54	*Passiflora luetzelburgii* Harms	*Passiflora*	Rio Pardo de Minas, Minas Gerais
55	*Passiflora malacophylla* Spruce ex Mast.	*Passiflora*	Rio das Ostras, Rio de Janeiro
56	*Passiflora maliformis* L.	*Passiflora*	Selection Embrapa, Distrito Federal
57	*Passiflora maliformis* L.	*Passiflora*	Boa Vista, Roraima
58	*Passiflora maliformis* L.	*Passiflora*	Guajará Mirim, Rondônia
59	*Passiflora mendoncaei* Harms	*Passiflora*	Monte Verde, Minas Gerais
60	*Passiflora micropetala* Mast.	*Decaloba*	Iranduba, Amazonas
61	*Passiflora miersii* Mast. in Mart.	*Passiflora*	Monte Verde, Minas Gerais
62	*Passiflora misera* Kunth	*Decaloba*	Trancoso, Bahia
63	*Passiflora morifolia* Mast. in Mart.	*Decaloba*	Lavras, Minas Gerais
64	*Passiflora mucronata* Lam.	*Passiflora*	Campos dos Goytacazes, Rio de Janeiro
65	*Passiflora nitida* Kunth	*Passiflora*	Presidente Figueiredo, Amazonas
66	*Passiflora nitida* Kunth	*Passiflora*	Planaltina, Distrito Federal
67	*Passiflora nitida* Kunth	*Passiflora*	Marabá, Pará
68	*Passiflora odontophylla* Harms ex Glaz.	*Passiflora*	Caeté, Minas Gerais
69	*Passiflora organensis* Gardn.	*Decaloba*	Serra dos Órgãos, Rio de Janeiro
70	*Passiflora pedata* L.	*Passiflora*	Manaus, Amazonas
71	*Passiflora picturata* Ker	*Passiflora*	Álter do Chão, Pará
72	*Passiflora pohlii* Mast. in Mart.	*Decaloba*	Planaltina, Distrito Federal
73	*Passiflora porophylla* Vell.	*Decaloba*	Caeté, Minas Gerais
74	*Passiflora quadrangularis* L.	*Passiflora*	Silvania, Goiás
75	*Passiflora quadrangularis* L.	*Passiflora*	Commercial Orchard
76	*Passiflora quadrifaria* Vanderpl.	*Distephana*	Manaus, Amazonas
77	*Passiflora quadriglandulosa* Rodschied	*Distephana*	Porto Velho, Rondônia
78	*Passiflora racemosa* Brot.	*Passiflora*	Búzios, Rio de Janeiro
79	*Passiflora recurva* Mast. in Mart.	*Passiflora*	Rio Pardo de Minas, Minas Gerais
80	*Passiflora rhamnifolia* Mast.	*Astrophea*	Caeté, Minas Gerais
81	*Passiflora riparia* Mart.	*Passiflora*	Confresa, Mato Grosso
82	*Passiflora rubra* L.	*Decaloba*	Monte Verde, Minas Gerais
83	*Passiflora saxicola* Gontsch.	*Decaloba*	Porto Seguro, Bahia
84	*Passiflora sclerophylla* Harms	*Astrophea*	Manaus, Amazonas
85	*Passiflora setacea* DC.	*Passiflora*	Tapiramutá, Bahia
86	*Passiflora setacea* DC.	*Passiflora*	Planaltina, Distrito Federal
87	*Passiflora setacea* DC.	*Passiflora*	Manhuaçu, Minas Gerais
88	*Passiflora setacea* DC.	*Passiflora*	Janaúba, Minas Gerais
89	*Passiflora sidaefolia* M. Roemer	*Passiflora*	Caparaó, Minas Gerais
90	*Passiflora speciosa* Gardn.	*Passiflora*	Manhuaçu, Minas Gerais
91	*Passiflora suberosa* L.	*Decaloba*	Macapá, Amapá
92	*Passiflora subrotunda* Mast. in Mart.	*Passiflora*	Natal, Rio Grande do Norte
93	*Passiflora tenuifila* Killip	*Passiflora*	Patos de Minas, Minas Gerais
94	*Passiflora tholozanii* Sacco	*Distephana*	Girau, Rondônia
95	*Passiflora tricuspis* Mast. in Mart.	*Decaloba*	Planaltina, Distrito Federal
96	*Passiflora triloba* Ruiz & Pav. ex DC.	*Passiflora*	Cruzeiro do Sul, Acre
97	*Passiflora trintae* Sacco	*Passiflora*	Rio Pardo, Minas Gerais
98	*Passiflora variolata* Poepp. & Endl.	*Distephana*	Manaus, Amazonas
99	*Passiflora vespertilio* L.	*Decaloba*	Manaus, Amazonas
100	*Passiflora villosa* Vell.	*Passiflora*	Alto Paraíso, Goiás
101	*Passiflora vitifolia* Kunth	*Passiflora*	Poconé, Mato Grosso

### Cross-species transferability of *P. edulis* microsatellite markers

In order to test the potential cross-species transferability of novel *P. edulis* microsatellite markers to other *Passiflora* species, we genotyped 90 accessions belonging to 78 *Passiflora* species native to Brazil (Table 1, rows 12 to 101), maintained by the Passion Fruit Germplasm Bank, Embrapa Cerrados, Planaltina, DF. These passion fruit species belong to four subgenera (*Astrophea*, *Decaloba*, *Distephana* and *Passiflora*). These accessions were genotyped with 18 polymorphic markers out of a sample of 60 markers initially selected for testing. Successful PCR amplifications were recorded as presence or absence of amplicons if the allele sizes were detected in the approximate expected range.

For most *Passiflora* species, only one accession was represented in the Germplasm Bank. However, for those species with two to five accessions available, cross-amplification and marker polymorphism could be computed (Table 1). Allele frequencies observed in 27 accessions of six species (*P. edulis, P. alata, P. maliformis, P. nitida, P. quadrangularis* and *P. setacea*) plus accession 11 (Table 1, BRS Maracujá Jaboticaba) were used to estimate pairwise genetic distances using the Band coefficient [50]. BRS Maracujá Jaboticaba is an autogamous variety of sour passion fruit of unknown phylogeny which produces small fruits of purple rind. Genetic similarities detected by microsatellite markers was explored by Principal Coordinate Analysis (PCoA) using NTSYSpc v.2.10 [51]. An analysis of population structure and ancestry of these 28 accessions based on Bayesian statistics, without prior assignment to species, was also performed using Structure v.2.3.4 [52, 53]. Batch runs with correlated and independent allele frequencies among inferred clusters were tested with population parameters set to admixture model (burn-in 250,000; run-length 500,000). In order to identify the number of clusters in the sample of *Passiflora* accessions, the values of ln P(D) were obtained for tests of K ranging from 1 to10 using 20 independent runs for each K (length of burnin period: 50,000; number of MCMC reps after burnin: 50,000). The most probable value of K for each test was detected by delta K [54]. *Passiflora* accessions were allocated to a cluster if Q values were greater or equal to 0.70, or otherwise considered as intermediate or admixed. DNA extraction and quantification of all passion fruit accessions followed the procedures described above.

*Microsatellite marker PCR assays -* Multiplex panels for simultaneous evaluation of microsatellite markers were designed using Multiplex Manager [55]. PCR assays were carried in a final volume of 5 μL containing 5 ηg of genomic DNA, 1X QIAGEN Multiplex PCR Kit Master Mix (QIAGEN), 0.5X Q-Solution (QIAGEN), and 0.2 μM of each primer. Reactions were performed on a Veriti™ Thermal Cycler (Applied Biosystems, USA) using the following amplification program: 95 °C for 15 min; 35 cycles at 94 °C for 30 s, 55, 57 or 60 °C for 90 s, and 72 °C for 60 s; followed by a final extension step at 60 °C for 60 min. We added 9 μL of Hi-Di™ Formamide (Applied Biosystems, USA) and a ROX-labeled internal size standard to 1 μL of the PCR product and denatured at 94 °C for 5 min. Denatured products were injected on an ABI3730 (Applied Biosystems, USA) automated sequencer. Allele size calling and genotyping were carried out with GeneMapper® v4.1 (Applied Biosystems, USA). Automated allelic binning was performed with Tandem [56]. Fisher’s exact test was used to test the association between the level of marker polymorphism and the repeat size (di- or tri-nucleotide) using the MedCalc Statistical Software v.12.7.7 [http://www.medcalc.org; 2013].

## Results

Partial sequencing and de novo assembly of the *Passiflora* genome for microsatellite site detection.

Sequence assembly was based on 225,293,527 short read DNA sequences (average length = 62.65 bp), representing 14.1 Gbp (Table 2), which corresponds to ~4.5× coverage of the passion fruit genome, assuming a genome size of 3126 Mbp [57]. A total of 234,239 contig segments showing variation in size from 166 to 45,662 bp, average size of 707 bp and covering 165,702,691 bp, were examined for the presence of microsatellite sites. The genome sequences of the *P. edulis* genome have been deposited in GenBank under the BioProject ID SUB2376276.

**Table 2:** Summary of Illumina paired-end read sequence data, de novo assembly and detection of microsatellite repeats in the *Passiflora edulis* genome

Sequence information	Total #	Size variation (bp)	Average length (bp)	Total (bp)
Illumina paired-end reads	225,293,527	52–76	62.65	14,113,860,125
Contigs	234,239	166–45,662	707	165,702,691
Microsatellite sequences	Total #	>5 repeats		
Compound and/or imperfect microsatellites	1,544,549	-		
Perfect di-nucleotides	360,162	13,391		
Perfect tri-nucleotides	60,669	1436		
Perfect tetra-nucleotides	7463	186		
Total	1,972,843	-		

A batch of 1,972,843 microsatellite sites matched the criteria set for simple sequence repeat discovery in the assembled contig segments (Table 2). Perfect microsatellite included 360,162 di-nucleotide repeats with the number of repeats ranging from 3 to 20 (13,391 > 5 repeats). Perfect tri-nucleotide repeats included 60,669 sites ranging from 3 to 14 repeats (1436 > 5 repeats). Perfect tetra-nucleotide repeats included 7463 sites ranging from 3 to 13 repeats (186 > 5 repeats).

Sequence analysis of *P. edulis* contigs allowed 37,761 gene annotations and identified 5947 sequence repeats located in putative coding regions, of which 2990 hits were non redundant. An ab initio prediction of coding regions resulted in the compilation of 101,361 hits in exon regions of the 47,706 scaffolds evaluated.

**Fig. 1:**
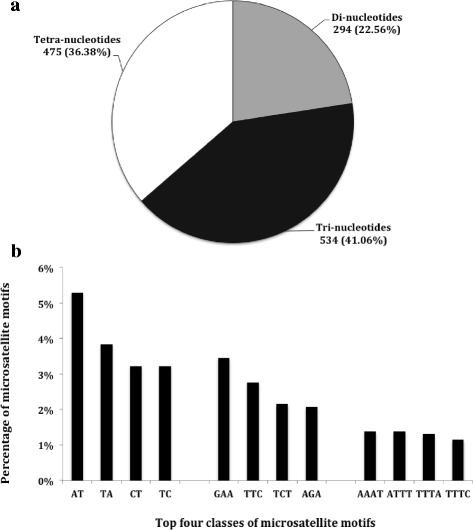
**a** Distribution of di-, tri-, and tetra-nucleotide perfect microsatellites on contigs with a minimum 15X average coverage; **b** Distribution of most frequent repeat motifs on contigs with a minimum 15X average coverage

Using a minimum 15X average coverage as a cut off, a total of 1300 perfect microsatellite sites were selected in functional and structural genomic regions of sour passion fruit. In this sample of microsatellite sites, tri-nucleotide repeats were the most abundant class (534 sites), followed by tetra-nucleotide (475) and di-nucleotide (294) (Fig. 1a). The most frequent types of microsatellite sequences observed on each class were AT/TA, GAA/TTC and AAAT/ATTT (Fig. 1b). The most frequent di-nucleotide repeat motif (AT) was also the most abundant one, comprising (5.3%) of the perfect microsatellite region detected on contigs with at least 15X coverage. On the other hand, tri- and tetra-nucleotide repeat motifs had a more balanced distribution among different classes.

The list of 1300 microsatellite sites was further examined for PCR primer development (Additional file 1). Primer pairs flanking the DNA repeats could be developed for 816 microsatellite sites, which were suitable for design within each contig, showing no adjacent simple sequence repeat loci and attending the minimal specified requirements which have been previously described. The new microsatellite markers were given the “BrPe” prefix. The list includes 149 di-, 329 tri- and 338 tetra-nucleotide markers. Approximately 56% of the markers are located in functional regions of the *P. edulis* genome (60 di-, 263 tri- and 139 tetra-nucleotide markers) and the remaining in structural regions.

**Fig. 2:**
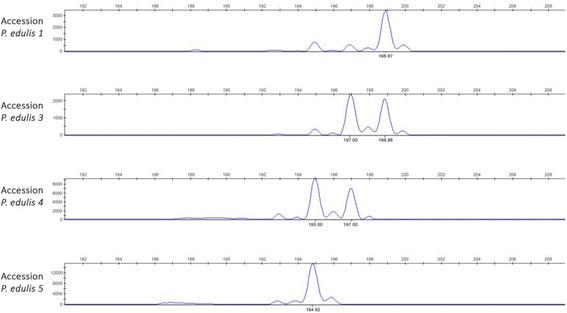
Electropherograms of marker BrPe0006 showing amplification patterns and DNA polymorphism between four accessions of *P. edulis* (accessions1, 3, 4, 5) (Y axis = pfu; X axis = allele size)

A random sample of 60 markers (50 di- and 10 tri-nucleotide repeats) was labeled with fluorescent dyes and combined for simultaneous amplification in duos or trios in order to test their genotyping efficiency and marker polymorphism on passion fruit accessions. We tested 25 panels, usually containing two markers each, for simultaneous allele amplification. A total of 52 markers could readily amplify PCR products in all 25 duo panels without any adjustment in PCR amplification conditions (Fig. 2). Five markers worked better in solo amplifications (BrPe0014, BrPe0021, BrPe0033, BrPe0042, BrPe0043). PCR amplicons were not obtained for only three markers (5%) (BrPe0004, BrPe0005, BrPe0048), although further attempts to adjust PCR were not pursued. This represents a very high rate of PCR amplification success for microsatellite markers.

### Descriptive statistics of microsatellite markers

Among the 57 markers which produced amplicons, 42 (~74%) were polymorphic when tested on a sample of ten *P. edulis* germplasm accessions, providing the detection of 137 alleles (Table 3). Fifteen markers were not polymorphic (nine di-nucleotide and six tri-nucleotide repeat markers) (Additional file 1). The number of observed alleles for all polymorphic microsatellite markers ranged from 2 to 7, with an average value of 3.26 alleles per locus (Table 3). Marker expected heterozigosity (He) values ranged from 0.19 to 0.84, with an average of 0.55. Observed heterozigosity (Ho) values ranged from 0.00 to 1.00, with an average of 0.35. Polymorphism Information Content (PIC) values ranged from 0.16 to 0.77, with an average of 0.45 (Table 3).

**Table 3:** Descriptive statistics of novel *Passiflora edulis* microsatellite markers

**#**	Marker	Primer Sequences 5′-3′	RepeatMotif	T_a_	AlleleN	Allele Size Range (bp)	He	Ho	PIC
1	BrPe0032	F:TTGCACAATGACCAATGTTGT R:CTGAGCACCTTGTCAAAATACA	(AT)_13_	60 °C	7	137–157	0.84	1.00	0.77
2	BrPe0028	F:CAAAAGGAACAGGGAAGA R:GAAAGAGAGAAAGACAGAGA	(TA)_6_	55 °C	5	90–110	0.80	0.50	0.72
3	BrPe0024	F:CCCTACCTTTCTCTGCTT R:CATCTCCTCTATCTCCTTC	(TC)_7_	55 °C	4	221–231	0.76	0.70	0.68
4	BrPe0031	F:AGGTCGGTGGGTGTGTTTAG R:CATTCAACTCCCCAAAAGGT	(TA)_9_	60 °C	5	134–150	0.77	0.67	0.67
5	BrPe0014	F:AATATGGCTGGGGAAAAC R:TTCCTGTCTTTGGACCTT	(AG)_7_	57 °C	5	215–227	0.75	0.50	0.67
6	BrPe0033	F:GCCATGAGAGACTTGGGAGA R:CGGTTGCCAAAAAGAAGAGA	(AT)_8_	60 °C	5	237–249	0.72	0.30	0.65
7	BrPe0038	F:TTTCAACTTTTCGTGTGTGC R:TGTTGTTGCTTGGAAGGATG	(AT)_6_	60 °C	5	154–176	0.73	0.60	0.64
8	BrPe0042	F:CATGCATTCATTTGTTTTTCTTG R:GATGCTGGGAAAAAGAGTGC	(AT)_8_	60 °C	6	142–160	0.71	0.80	0.63
9	BrPe0003	F:CTTTCTCTCCCTATACCC R:CCCTCCATAATCACATAAC	(TC)_11_	55 °C	5	277–291	0.70	0.40	0.62
10	BrPe0043	F:TCATACATGGATGTCAAATCGATAC R:GCGGACCAAGAAAATTCAAA	(AT)_8_	60 °C	4	199–207	0.71	0.50	0.60
11	BrPe0006	F:AAGGAAAAGAACAGCCTCA R:CGCTCTCAAATCAGTCAAA	(TC)_10_	55 °C	4	193–199	0.68	0.40	0.59
12	BrPe0002	F:AAAGCCCAGATGAAGTGAA R:GGCTCCAATCAGAAGTGT	(AG)_12_	55 °C	3	177–185	0.69	0.56	0.58
13	BrPe0021	F:ACTTCCTCATCATTCG R:GCTATGCCTCTTTTTG	(TA)_7_	55 °C	3	158–164	0.67	0.25	0.56
14	BrPe0036	F:TCGGACCTTAAAACCGAGAA R:CAGCACCAAAATTTGACGAG	(TC)_6_	60 °C	4	197–203	0.65	0.10	0.54
15	BrPe0023	F:AGATACCACACCCAATAG R:TTGGAGTTGTTGGGGA	(CT)7	55 °C	6	118–132	0.57	0.40	0.52
16	BrPe3011	F:CCGGTCTTCCTGATTGACTC R:CCTCTCTCACCTGGAACTGC	(TTC)_4_	60 °C	3	157–163	0.62	0.30	0.50
17	BrPe0037	F:TGATAATGCAGCGAAAGAGC R:TCACACTCCATTTGCTCTGC	(TG)6	60 °C	3	227–231	0.61	0.20	0.49
18	BrPe0010	F:GAAGAAAAAAGGGCTTG R:GTTAGGGTTTGGAGGA	(TC)_9_	55 °C	3	200–204	0.60	0.40	0.48
19	BrPe0001	F:GTTGAGAGGATTGTGTTTG R:ATGGTAGAGGAGGAGAGA	(CT)_14_	55 °C	3	143–157	0.56	0.14	0.46
20	BrPe0012	F:AGAGAGAGAGAGAGAG R:ACATCATACTCCTCATCC	(AG)8	55 °C	3	214–218	0.58	0.13	0.45
21	BrPe0008	F:TTTTCAGCCTCCACTCTT R:TACACCACCAACACTCAC	(AG)9	55 °C	3	264–274	0.57	1.00	0.44
22	BrPe0025	F:CAAGGAACCAGAACAAGAAGAA R:GAAGAACAAGCCAGCCCA	(GA)6	55 °C	3	114–126	0.57	0.11	0.44
23	BrPe0039	F:GCTGCTCCACTGTGAATGTC R:AACCTAGCCCCGTCACAGTA	(AT)6	60	3	193–203	0.57	0.10	0.44
24	BrPe0050	F:TCAAGGGTATCTTTGGTGCTG R:AGCTTCAGCGAGACAAAACC	(TG)7	60 °C	3	197–205	0.56	0.20	0.44
25	BrPe0013	F:GATCGAGGTGAGGTACTG R:GGTTTGGCTTTAATGGAGG	(AG)8	55 °C	2	169–171	0.53	0.00	0.38
26	BrPe0020	F:TAAAGCATCAGGTCAG R:TAGATAGATTTGACGGG	(GT)7	55 °C	2	295–297	0.53	0.00	0.38
27	BrPe0034	F:CCTGTGGTGAAAATGGAACC R:GAGCCCTGGACTGACACATT	(CT)_15_	60 °C	2	217–227	0.56	1.00	0.38
28	BrPe0049	F:GGGAATCAAAACCATGCAGT R:CTCCCAGCTTCCACTCACTC	(TA)9	60 °C	2	189–191	0.53	0.11	0.38
29	BrPe3012	F:CGCCCTTTCTGAAGATAATCC R:GCAATGCTAAGAAGGCCAAG	(TCT)4	60 °C	2	181–183	0.53	0.11	0.38
30	BrPe0018	F:TCCTTCCTTCTCCTCC R:ACACTTGTCTCTCATCT	(CT)7	55 °C	3	135–149	0.43	0.30	0.37
31	BrPe0022	F:GGCATAGAAGTGGAAGGG R:GGAAGGGAAGTGAAGGGA	(AG)7	55 °C	2	98–104	0.51	0.20	0.37
32	BrPe0047	F:TGGGCCATTTCTTTTCTCTC R:GAATCCTGCATGAGTTGAGGA	(CT)9	60 °C	2	186–192	0.48	0.30	0.35
33	BrPe3014	F:CGGAAGCGTGCTCATAAAGT R:AAGCCTGTGAGGTTGATTCG	(AGA)5	60 °C	2	218–220	0.48	0.30	0.35
34	BrPe0007	F:AAAGCCCAGATGAAGTGAA R:GGCTCCAATCAGAAGTGT	(AG)9	55 °C	2	177–179	0.40	0.50	0.31
35	BrPe0027	F:TCCAATCTTCTCAACC R:CAAACTAGTAAACCCC	(TA)6	46 °C	3	97–101	0.35	0.20	0.30
36	BrPe3027	F:CCAAAATGCCCAAAATGTCT R:GTCCGTGAGGAGATGTCGAT	(GGT)4	60 °C	3	178–202	0.35	0.40	0.30
37	BrPe0019	F:AAAGAGAAGGATGGATG R:AAAAAGGACGAGGAAGA	(TC)7	55 °C	2	210–214	0.36	0.14	0.28
38	BrPe0044	F:GGACGCTAAGAGACCCATTG R:TAAAAGCCCCACTTGCAATC	(TA)6	60 °C	2	217–219	0.33	0.38	0.26
39	BrPe0016	F:TGGTTGGTGGGTCTTGT R:CTCTTTCCTCTCTCTCTCTCT	(AG)7	55 °C	2	277–279	0.21	0.22	0.18
40	BrPe0045	F:CGCTTCCACTTTACCAGCTC R:GACCAACAACAGGCACAATG	(GT)8	60 °C	2	183–185	0.21	0.22	0.18
41	BrPe0011	F:GTTCTACTCCCTCATT R:CTTCTTAACATCCCCA	(CT)8	53 °C	2	74–80	0.19	0.20	0.16
42	BrPe0017	F:TTGTCTCTCGGTTCTCT R:CAAACACAAAACCCCC	(AG)7	55 °C	2	86–90	0.19	0.00	0.16
	AVERAGE				3.26		0.55	0.35	0.45

F: forward primer; R: reverse primer; T_a_: annealing temperature; Ho: observed heterozygosity; He: expected heterozygosity; PIC: polymorphic information content

We checked whether the size ranges for the polymorphic loci included their expected product size on *P. edulis*. Expected product sizes for each microsatellite marker are based on sequence information generated by the de novo assembly process. The proportion of markers that generated amplicons within 5% of their expected sizes was 100% (42 out of 42). Approximately 55% of the polymorphic markers generated amplicons with product size exactly as expected (23 out of 42).

Out of 50 di-nucleotide markers tested for DNA polymorphism, 17 were located on structural genomic regions and 33 on putative functional sites of the *P. edulis* genome (Additional file 1). We found no significant association (Fisher’s exact test *p*-value = 0.64) between the level of marker polymorphism and repeat size (di- or tri-nucleotide).

Microsatellite marker cross-amplification in *Passiflora* species.

Markers were ranked by PIC values and used to evaluate their cross amplification in 79 *Passiflora* species (including *P. edulis*). The average PIC value for the 18 selected markers was 0.60, varying from 0.46 to 0.77 (Table 3, markers 1–16, 18, 19). A survey on the potential cross-amplification of these microsatellite markers in a collection of *Passiflora* species showed that 72% of the marker/species combinations resulted in positive amplifications (Table 4)*,* with cross-amplification values ranging from 33% to 94%. Such a large proportion of marker transferability was not anticipated. Three markers produced PCR products in all 79 *Passiflora* species (BrPe0032, BrPe0038, BrPe3011). BrPe0032 had the highest PIC and number of alleles in the tested sample of *P. edulis* accessions. Primers BrPe0001, BrPe0034 and BrPe0042 also worked in most of the species tested, with the exception of *P. porophylla* (BrPe0001), *Passiflora triloba* and *P. vitifolia* (BrPe0034), and *P. capsularis* and *P. gibertii* (BrPe0042). Interestingly, at least 14 markers (BrPe0032, BrPe0038, BrPe3011, BrPe0001, BrPe0034, BrPe0042, BrPe0036, BrPe0006, BrPe0010, BrPe0028, BrPe0031, BrPe0021, BrPe0003, BrPe0033) could cross amplify PCR products in 17 species (*P. cerasina, P. coccinea, P. decaisneana, P. quadrangularis, P. riparia, P. variolata, P. mendoncaei, P. nitida, P. racemosa, P. recurva, P. ligularis, P. maliformis, P. odontophylla, P. pedata, P. tenuifila, P. alata, P. setacea).* Fifty percent of the markers produced amplicons in all but two of the species tested, *P. pohlii* (*Decaloba*) and *P. sclerophylla* (*Astrophea*).

**Table 4:** Cross-species transferability of 18 *P. edulis* microsatellite markers to 78 *Passiflora* species

Species	BrPe0043	BrPe0014	BrPe0002	BrPe0024	BrPe0033	BrPe0003	BrPe0021	BrPe0031	BrPe0028	BrPe0006	BrPe0010	BrPe0036	BrPe0034	BrPe0042	BrPe0001	BrPe0032	BrPe0038	BrPe3011	%
*Passiflora pohlii*	−	−	−	−	−	−	−	−	−	−	−	−	+	+	+	+	+	+	33%
*Passiflora sclerophylla*	−	−	−	−	+	−	−	−	+	−	−	−	+	+	+	+	+	+	44%
*Passiflora suberosa*	−	−	−	−	−	−	−	−	+	+	+	−	+	+	+	+	+	+	50%
*Passiflora eichleriana*	−	−	−	−	+	−	+	−	−	+	+	−	+	+	+	+	+	+	56%
*Passiflora hatschbachii*	−	−	−	−	+	+	−	+	−	−	−	+	+	+	+	+	+	+	56%
*Passiflora picturata*	−	−	−	−	−	−	−	−	+	+	+	+	+	+	+	+	+	+	56%
*Passiflora porophylla*	−	+	−	−	−	−	−	+	+	+	−	+	+	+	−	+	+	+	56%
*Passiflora saxicola*	−	−	−	−	−	−	−	+	+	−	+	+	+	+	+	+	+	+	56%
*Passiflora tricuspis*	−	−	−	−	−	+	−	−	+	+	−	+	+	+	+	+	+	+	56%
*Passiflora araujoi*	−	−	−	−	−	−	+	+	+	+	−	+	+	+	+	+	+	+	61%
*Passiflora auriculata*	−	−	−	−	−	−	−	+	+	+	+	+	+	+	+	+	+	+	61%
*Passiflora edmundoi*	−	−	−	−	−	+	−	−	+	+	+	+	+	+	+	+	+	+	61%
*Passiflora ferruginea*	−	−	−	+	−	−	+	+	+	−	−	+	+	+	+	+	+	+	61%
*Passiflora haematostigma*	−	−	−	+	+	−	−	−	+	+	−	+	+	+	+	+	+	+	61%
*Passiflora loefgrenii*	−	−	−	+	−	−	+	−	−	+	+	+	+	+	+	+	+	+	61%
*Passiflora micropetala*	−	−	−	+	−	−	−	+	+	−	+	+	+	+	+	+	+	+	61%
*Passiflora misera*	−	−	−	+	−	−	+	−	+	+	+	−	+	+	+	+	+	+	61%
*Passiflora morifolia*	−	−	−	−	−	+	+	−	−	+	+	+	+	+	+	+	+	+	61%
*Passiflora rubra*	−	+	−	+	−	−	−	−	+	+	−	+	+	+	+	+	+	+	61%
*Passiflora vespertilio*	−	−	−	−	−	−	−	+	+	+	+	+	+	+	+	+	+	+	61%
*Passiflora acuminata*	−	−	−	−	−	−	+	+	+	+	+	+	+	+	+	+	+	+	67%
*Passiflora caerulea*	−	−	+	−	−	−	+	−	+	+	+	+	+	+	+	+	+	+	67%
*Passiflora capsularis*	+	−	−	+	−	−	+	+	−	+	+	+	+	−	+	+	+	+	67%
*Passiflora cervii*	−	−	−	+	−	−	−	+	+	+	+	+	+	+	+	+	+	+	67%
*Passiflora jilekii*	−	−	−	+	−	−	+	+	+	+	+	−	+	+	+	+	+	+	67%
*Passiflora rhamnifolia*	−	−	−	−	+	−	−	+	+	+	+	+	+	+	+	+	+	+	67%
*Passiflora tholozanii*	−	−	−	−	−	+	−	+	+	+	+	+	+	+	+	+	+	+	67%
*Passiflora triloba*	−	+	−	−	+	+	+	−	+	+	+	−	−	+	+	+	+	+	67%
*Passiflora vitifolia*	+	−	−	−	+	−	−	+	+	+	+	+	−	+	+	+	+	+	67%
*Passiflora ambigua*	+	−	−	+	−	−	+	−	+	+	+	+	+	+	+	+	+	+	72%
*Passiflora biflora*	−	−	−	−	−	+	+	+	+	+	+	+	+	+	+	+	+	+	72%
*Passiflora boticarioana*	−	−	+	+	+	+	−	−	−	+	+	+	+	+	+	+	+	+	72%
*Passiflora cerradense*	−	−	−	+	+	+	−	−	+	+	+	+	+	+	+	+	+	+	72%
*Passiflora chlorina*	+	−	−	+	+	+	−	−	−	+	+	+	+	+	+	+	+	+	72%
*Passiflora cincinnata*	−	−	−	+	+	+	−	−	+	+	+	+	+	+	+	+	+	+	72%
*Passiflora elegans*	−	−	+	−	+	+	−	+	−	+	+	+	+	+	+	+	+	+	72%
*Passiflora foetida*	−	−	−	−	+	+	+	+	−	+	+	+	+	+	+	+	+	+	72%
*Passiflora gardneri*	−	−	+	+	+	+	+	−	−	−	+	+	+	+	+	+	+	+	72%
*Passiflora gibertii*	−	−	−	+	+	−	+	+	+	+	+	+	+	−	+	+	+	+	72%
*Passiflora incarnata*	+	−	−	−	−	−	+	+	+	+	+	+	+	+	+	+	+	+	72%
*Passiflora junqueirae*	−	−	+	−	+	−	+	+	+	−	+	+	+	+	+	+	+	+	72%
*Passiflora kermesina*	−	+	+	−	−	+	−	+	+	−	+	+	+	+	+	+	+	+	72%
*Passiflora organensis*	−	−	−	−	−	+	+	+	+	+	+	+	+	+	+	+	+	+	72%
*Passiflora quadrifarial*	−	−	−	−	+	+	−	+	+	+	+	+	+	+	+	+	+	+	72%
*Passiflora trintae*	−	−	−	+	−	+	+	−	+	+	+	+	+	+	+	+	+	+	72%
*Passiflora villosa*	−	−	+	−	+	+	−	+	−	+	+	+	+	+	+	+	+	+	72%
*Passiflora actinia*	−	−	+	−	+	+	+	−	+	+	+	+	+	+	+	+	+	+	78%
*Passiflora amethystina*	−	−	+	+	+	−	+	−	+	+	+	+	+	+	+	+	+	+	78%
*Passiflora cerasina*	−	−	−	−	+	+	+	+	+	+	+	+	+	+	+	+	+	+	78%
*Passiflora coccinea*	−	−	−	−	+	+	+	+	+	+	+	+	+	+	+	+	+	+	78%
*Passiflora decaisneana*	−	−	−	−	+	+	+	+	+	+	+	+	+	+	+	+	+	+	78%
*Passiflora galbana*	−	−	+	+	−	+	+	+	+	−	+	+	+	+	+	+	+	+	78%
*Passiflora hypoglauca*	−	−	+	−	+	+	−	+	+	+	+	+	+	+	+	+	+	+	78%
*Passiflora malacophylla*	−	−	+	+	+	+	+	−	−	+	+	+	+	+	+	+	+	+	78%
*Passiflora miersii*	−	−	+	−	+	+	+	+	+	−	+	+	+	+	+	+	+	+	78%
*Passiflora mucronata*	−	−	−	+	+	+	+	+	−	+	+	+	+	+	+	+	+	+	78%
*Passiflora quadrangularis*	−	−	−	−	+	+	+	+	+	+	+	+	+	+	+	+	+	+	78%
*Passiflora quadriglandulosa*	+	−	−	−	+	+	+	+	+	+	+	−	+	+	+	+	+	+	78%
*Passiflora riparia*	−	−	−	−	+	+	+	+	+	+	+	+	+	+	+	+	+	+	78%
*Passiflora sidaefolia*	−	−	−	+	+	+	−	+	+	+	+	+	+	+	+	+	+	+	78%
*Passiflora subrotunda*	−	−	−	+	+	−	+	+	+	+	+	+	+	+	+	+	+	+	78%
*Passiflora variolata*	−	−	−	−	+	+	+	+	+	+	+	+	+	+	+	+	+	+	78%
*Passiflora bahiensis*	+	−	−	+	−	+	+	+	+	+	+	+	+	+	+	+	+	+	83%
*Passiflora luetzelburgii*	−	−	+	+	+	+	+	−	+	+	+	+	+	+	+	+	+	+	83%
*Passiflora mendoncaei*	−	−	+	−	+	+	+	+	+	+	+	+	+	+	+	+	+	+	83%
*Passiflora nitida*	−	+	−	−	+	+	+	+	+	+	+	+	+	+	+	+	+	+	83%
*Passiflora racemosa*	−	−	+	−	+	+	+	+	+	+	+	+	+	+	+	+	+	+	83%
*Passiflora recurva*	−	−	+	−	+	+	+	+	+	+	+	+	+	+	+	+	+	+	83%
*Passiflora speciosa*	+	+	−	−	+	+	+	−	+	+	+	+	+	+	+	+	+	+	83%
*Passiflora glandulosa*	+	+	−	+	−	+	+	+	+	+	+	+	+	+	+	+	+	+	89%
*Passiflora laurifolia*	+	−	+	+	−	+	+	+	+	+	+	+	+	+	+	+	+	+	89%
*Passiflora ligularis*	−	−	+	+	+	+	+	+	+	+	+	+	+	+	+	+	+	+	89%
*Passiflora maliformis*	−	−	+	+	+	+	+	+	+	+	+	+	+	+	+	+	+	+	89%
*Passiflora odontophylla*	−	+	−	+	+	+	+	+	+	+	+	+	+	+	+	+	+	+	89%
*Passiflora pedata*	−	−	+	+	+	+	+	+	+	+	+	+	+	+	+	+	+	+	89%
*Passiflora tenuifila*	−	−	+	+	+	+	+	+	+	+	+	+	+	+	+	+	+	+	89%
*Passiflora alata*	−	+	+	+	+	+	+	+	+	+	+	+	+	+	+	+	+	+	94%
*Passiflora setacea*	−	+	+	+	+	+	+	+	+	+	+	+	+	+	+	+	+	+	94%
*Passiflora edulis*	+	+	+	+	+	+	+	+	+	+	+	+	+	+	+	+	+	+	100%

**Fig. 3:**
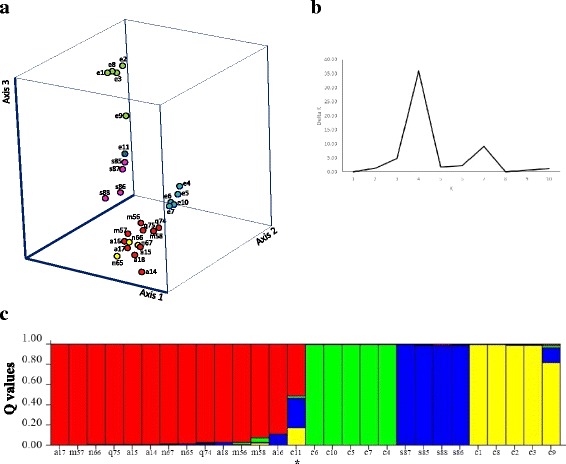
**a** Principal coordinates analysis of 28 accessions of Passiflora belonging to six closely related passion fruit species based on microsatellite polymorphism. Samples are identified according to accession number on Table 1, preceded by species initial: *P. edulis* (samples e1 to e11); *P. alata* (samples a14 to a18); *P. maliformis* (samples m56 to m58); *P. nitida* (samples n65 to n67); *P. quadrangularis* (samples q74 and q75) and *P. setacea* (samples s85 to s88); **b** Plot of K vs Delta K values to define the most probable number of clusters in the analysis of population structure and ancestry of 28 Passiflora accessions without prior assignment to species; **c** Passiflora accessions were allocated to clusters based on Q values (Q > 0.70) for K = 4. Admixed or intermediate samples identified with an asterisk

The new microsatellite markers uncovered genetic diversity in *P. edulis* (Fig. 2, Table 3) and also in other related species (Fig. 3). PCoA analysis based on marker polymorphism assessed by 18 markers on 28 accessions belonging to six species (*P. edulis, P. alata, P. maliformis, P. nitida, P. quadrangularis* and *P. setacea*) allowed their separation in four main clusters. The variation captured by eigenvalue of the first three axis was high (axis 1 = 32.08%, axis 2 = 14.20% and axis 3 = 11.10%). Interestingly, the *P. edulis* accessions formed two clusters (Fig. 3a) and could be easily separated from the accessions of the other *Passiflora* species. The only exception was BRS Maracujá Jaboticaba (Table 1, accession 11), which did not cluster with the two *P. edulis* groups and seems to be closely associated to a cluster formed by *P. setacea* accessions. The fourth cluster included accessions of *P. nitida, P. quadrangularis, P. alata* and *P. maliformis*. Although the accessions of these four species could be discriminated with this set of microsatellite markers, they were all included in the same cluster. An analysis of population structure and ancestry of these 28 accessions with no prior assignment of species also inferred the existence of four main clusters, estimated by plotting values of K vs Delta K, for K varying from 1 to 10 (Fig. 3b). Again the accessions of *P. edulis* were allocated to two clusters, *P. setacea* accessions were separated in a third group, while accessions of *P. nitida, P. quadrangularis, P. alata* and *P. maliformis* formed a fourth group. All accessions were allocated to one of the four clusters with Q value ≥70, with the exception of BRS Maracujá Jaboticaba (accession e11), which showed an admixed or intermediate profile.

## Discussion

Most microsatellite markers of *P. edulis* and other *Passiflora* species developed so far were obtained by sequencing of genomic libraries enriched with simple sequence repeat regions [17–19, 23, 30–32]. There are only ~200 microsatellite di-nucleotide markers available for *P. edulis* [17, 19, 23]. Here we describe the efficient use of NGS to obtain a large amount of sequence data and applied bioinformatics tools to develop a novel sample of 816 microsatellite markers for this species. The lack of a significant set of polymorphic microsatellite markers for *P. edulis* and the majority of the *Passiflora* species was one of the main justifications of the present study. Microsatellite marker technology is used routinely in many genetic and breeding applications in different organisms, but it has had very limited use in passion fruit research. Other marker technologies, such as Single Nucleotide Polymorphism (SNP), have recently become accessible to several plant species and should soon be also available for sour passion fruit.

It has been observed that most microsatellite markers developed for *P. edulis* usually detect low polymorphism, estimated as varying from 15% [19] to 24.7% [23]. These results have been interpreted as evidence that genetic diversity in *P. edulis* is low [18, 19], contrasting with the high morphological [7] and agronomic diversity [8, 10] observed in this species. In order to verify how polymorphic is the new set of microsatellite markers, we tested a random sample of 60 new markers on ten accessions of *P. edulis* collected in different regions of Brazil and estimated genetic parameters such as Ho, PIC and number of alleles. Approximately 74% of the di- and tri-nucleotide markers with amplicon products were polymorphic, and PIC, Ho and allele number were high. PIC values for 80.9% (38/47) of the di-nucleotides markers ranged from 0.26 to 0.77, and for 40% (4/10) of the tri-nucleotides markers from 0.30 to 0.50. Using DNA fingerprinting based on only two markers (BrPe0028 and BrPe0032), one could discriminate all *P. edulis* accessions used in the present study. These estimates are similar to values found for other allogamous species where NGS technology was used for microsatellite development, such as the forage *Brachiaria ruziziensis* [39] or radish *Raphanus sativus* [58]*.* Therefore, we did not find evidence of low microsatellite polymorphism in *P. edulis* as assessed by the new set of microsatellite markers. Quite contrary, the majority of the markers tested were highly polymorphic. It is possible that the low polymorphism in *P. edulis* assessed by previous studies with microsatellite markers was actually caused by hidden genetic relatedness of passion fruit samples used in the screening, or simply because the markers tested were located in more conserved regions of the sour passion fruit genome.

Perfect microsatellite markers represent only a small fraction (~10%) of the total number of *P. edulis* markers available so far. The vast majority are compound or imperfect motif markers, which are hard to interpret on routine genotyping assays due to allele binning difficulties [26, 27]. Also, most of the studies with *P. edulis* microsatellite markers were based on allelic discrimination in agarose gels [59, 60] or polyacrylamide gels [16, 17, 19, 61, 62], what added more challenge to the analysis of compound and imperfect microsatellite markers. This could be a constraint to some applications, especially for population genetic studies [28]. All new markers are based on repeat of the same nucleotide motif without interruption or variation, what should facilitate genetic analysis.

We tested the new set of *P. edulis* microsatellite markers on other 78 *Passiflora* species. The percentage of cross-species transferability to other species of the subgenus *Passiflora* was high (75.4%), similar to *Distephana* (71.11%). However, it decreased to species belonging to *Astrophea* (63.33) and *Decaloba* (59.72%). Oliveira et al. [24] obtained similar results for cross-species transferability of *P. edulis* microsatellite markers to subgenera *Passiflora* (>73%) and *Decaloba* (54%). It is interesting to notice that *P. edulis* PCR products were obtained for at least 50% of the tested markers in all 90 accessions of other *Passiflora* species, with the exception of *P. pohlii* (*Decaloba*) and *P. sclerophylla* (*Astrophea*). This is an indication that a substantial proportion of the new *P. edulis* microsatellite markers can potentially be used in genetic studies of a great range of *Passiflora* species.

A combined analysis of 28 germplasm accessions of six *Passiflora* species (*P. edulis, P. alata, P. maliformis, P. nitida, P. quadrangularis* and *P. setacea*) using the new microsatellite markers demonstrated their efficiency to uncover genetic diversity in passion fruit. *P. edulis* accessions formed two clusters that could be easily separated from the accessions of the other *Passiflora* species. These *P. edulis* accessions were obtained in different regions of Brazil but there was no correlation between genetic clustering and geographic origin (data not shown). One of the clusters, however, is comprised of sour passion fruit accessions (Table 1, accessions 1–3 and 8) that have been widely used commercially (ex. accessions Maguary, CPGA1 and CPMSC1) and possibly derived from population of common ancestry [23]. Accession 8 (Criciúma, Santa Catarina) was originally collected in area close to *Passiflora* orchards, and its fruits might have been derived from cross-pollination with commercial cultivars. Although classified as *P. edulis*, the accession BRS Maracujá Jaboticaba (Table 1, accession 11) did not cluster with accessions of the two *P. edulis* groups. BRS Maracujá Jaboticaba seems to be closely associated to a third cluster formed by *P. setacea* accessions (Fig. 3a), although the estimated probability of inclusion in this group was not high (Q value = 0.52) (Fig. 3c). Recent analysis of the BRS Maracujá Jaboticaba mating system indicates that this accession is preferentially autogamous, while most *P. edulis* accessions are allogamous [63], what could explain its genetic distance to other sour passion fruit accessions. Further analysis on the role of different mating systems and mating plasticity in *P. edulis* genetic diversity should be pursued.

The fourth cluster included accessions of *P. nitida, P. quadrangularis, P. alata* and *P. maliformis*. Molecular phylogeny analysis of *Passiflora* species using nrITS, trnL-trnF and rps4 polymorphism grouped *P. alata*, *P. quadrangularis, P. maliformis*, *P. setacea* and *P. edulis* [64]. Plastid DNA analysis also found that *P. alata*, *P. nitida*, *P. edulis* and *P. maliformis* are closely related [65]. Paiva et al. [60] using microsatellites markers of Oliveira [23] and Pádua et al. [31] identified molecular similarity among *P. edulis* and *P. setacea*. *Passiflora* phylogeny is indeed very complex, with more than 520 species distributed in several continents. Microsatellite markers might help to understand genetic relationships within species and among accessions of closely related species.

Anthropic pressure at the centers of diversity is contributing to genetic erosion of many plant species, including *Passiflora* [66–68]. Intensive in situ conservation of native flora as well as efforts to collect wild species, landraces and local varieties for ex situ conservation are necessary for current and future use of passion fruit. Short term seed viability remains an important constraint to conservation [69, 70] and most collections rely on vegetative propagation for storage. It is a challenge to keep large numbers of passion fruit accessions by vegetative propagation of germplasm collections with usually restricted human and economic resources. Since vegetative propagation is the main form of conservation, each accession of passion fruit is usually comprised of one or a few plants per species or variety, imposing limits to ex situ genetic diversity storage. Routine activities of germplasm conservation and breeding demand the application of genome technology, including microsatellite markers, in conservation and use of passion fruit genetic resources.

## Conclusion

NGS technology was used to obtain a large amount of sequence data, which was applied to the development of hundreds of microsatellite markers for *P. edulis*. The new markers detected high levels of DNA polymorphism in *P. edulis* and could be used to assess genetic diversity in sour passion fruit accessions and in closely related species. The levels of cross-species transferability varied from 33% to 89% after testing 78 *Passiflora* species belonging to four subgenera (*Passiflora, Distephana, Astrophea* and *Decaloba*), indicating that a great number of *P. edulis* microsatellite markers could be potentially used in genetic analysis of other *Passiflora* species. This new set of microsatellite markers has many applications to germplasm conservation, breeding programs and genetic studies of passion fruit.

## Additional file

Additional file 1: Contig sequence information, repeat motifs, primer sequences, melting temperatures and expected product sizes of 816 di-, tri- and tetra-nucleotide microsatellite markers developed for the sour passion fruit (*Passiflora edulis* L.). (XLSX 244 kb)

## Abbreviations

AFLP: Amplified Fragment Length Polymorphism
BLAST: Basic Local Alignment Search Tool
CTAB: Cetyl Trimethylammonium Bromide
DNA: Deoxyribonucleic Acid
He: Expected Heterozigosity
Ho: Observed Heterozigosity
ISSR: Inter Simple Sequence Repeat
MCMC: Markov Chain Monte Carlo
NGS: Next-Generation Sequencing
nrITS: Nuclear Ribosomal Internal Transcribed Spacer
PCoA: Principal Coordinate Analysis
PCR: Polymerase Chain Reaction
PIC: Polymorphic Information Content
RAPD: Random Amplified Polymorphic DNA
SNP: Single Nucleotide Polymorphism
SOAP: Short Oligonucleotide Alignment Program
